# Supplementary material for: Inducing novel endosymbioses by implanting bacteria in fungi
Source: Nature. 2024 Oct 2;635(8038):415–22. doi: 10.1038/s41586-024-08010-x (PMC11560845; doi:10.1038/s41586-024-08010-x)
Supplement: Supplementary file 1 — Supplementary Tables 1–3. [file 41586_2024_8010_MOESM1_ESM.pdf]

nature portfolio

https://doi.org/10.1038/s41586-024-08010-x

Supplementary information

Inducing novel endosymbioses by implanting bacteria in fungi

In the format provided by the authors and unedited

SI Table 1: Strain List

Strain	In text name
R. microsporus van Tieghem ATCC62417	*R. microsporus* strain EH
R. microsporus var. *chinensis* (Saito) CBS 631.82	*R. microsporus* strain NH
E. coli BW25113 pDGUV-GFP	*E. coli*
M. rhizoxinica HKI454 pBBR_P12_GFP	*M. rhizoxinica*

SI Table 2: List of Reagents

Reagent	Supplier
Carbenicillin	PanReac AppliChem, USA
Ciprofloxacin	Merck, USA
Chloramphenicol	Roth, Germany
Gentamicin	PanReac AppliChem, USA
LB	ThermoFisher, USA
Agar	ThermoFisher, USA
Yeast extract	ThermoFisher, USA
Glycerol	VWR, USA
Potato Dextrose Broth (PDB)	Merck, USA
Dipotassium phophate	Roth, Germany
Potassium phosphate	Merck, USA
Sodium Citrate dihydrate	Fluka, USA
Sigmacote	Merck, USA
$(NH_4)_2SO_4$	Merck, USA
$MgSO_4$•7 H2O	Merck, USA
NaCl	Fluka, USA
D-Glucose	Merck, USA
Peptone	Merck, USA
Mannitol	Fluka, USA
Maleate	Fluka, USA
Onozuka R-10	Duchefa, Netherlands
Chitinase	Merck, USA
Sorbitol	ThermoFisher, USA
Tween20	NationalDiagnostics, USA
Sodium Acetate	Roth, Germany
Glacial Acetic Acid	VWR, USA
Ciliobrevin D	Merck, USA

SI Table 3: ID key for genomic samples ENA PRJEB76713

Study		ERP161218	ERA30640247	Description
Sample	G4_01	ERS20276168	SAMEA115755588	HKI-0454 starting material «B_{Anc}»
Sample	G4_02	ERS20276169	SAMEA115755589	HKI-0454 Round 10 Line 2
Sample	G4_03	ERS20276170	SAMEA115755590	HKI-0454 Round 10 Line 4
Sample	G4_04	ERS20276171	SAMEA115755591	HKI-0454 Round 10 Line 7
Sample	G4_05	ERS20276172	SAMEA115755592	HKI-0454 Round 10 Pooled
Sample	G4_06	ERS20276173	SAMEA115755593	HKI-0454 Round 7 Line 1
Sample	G4_07	ERS20276174	SAMEA115755594	HKI-0454 Round 7 Line 2
Sample	G4_08	ERS20276175	SAMEA115755595	HKI-0454 Round 7 Line 3
Sample	G4_09	ERS20276176	SAMEA115755596	HKI-0454 Round 7 Line 4
Sample	G4_10	ERS20276177	SAMEA115755597	HKI-0454 Round 7 Line 5
Sample	G4_11	ERS20276178	SAMEA115755598	HKI-0454 Round 7 Line 6
Sample	G4_12	ERS20276179	SAMEA115755599	HKI-0454 Round 7 Line 7
Sample	G4_13	ERS20276180	SAMEA115755600	HKI-0454 Round 7 Line 8

Sample	G4_14	ERS20276181	SAMEA115755601	HKI-0454 Round 7 Line 9
Sample	G4_15	ERS20276182	SAMEA115755602	HKI-0454 Round 7 Line 10
Sample	G4_16	ERS20276183	SAMEA115755603	HKI-0454 Round 3 Line 4
Sample	G4_17	ERS20276184	SAMEA115755604	HKI-0454 Round 4 Line 4
Sample	G4_18	ERS20276185	SAMEA115755605	HKI-0454 Round 5 Line 4
Sample	G4_19	ERS20276186	SAMEA115755606	HKI-0454 Round 6 Line 4
Sample	G4_20	ERS20276187	SAMEA115755607	HKI-0454 Round 7 Line 4
Sample	G4_21	ERS20276188	SAMEA115755608	HKI-0454 Round 8 Line 4
Sample	G4_22	ERS20276189	SAMEA115755609	HKI-0454 Round 9 Line 4
Sample	GG4	ERS20276190	SAMEA115755610	HKI-0454 Round 2 Line 1
Sample	GG5	ERS20276191	SAMEA115755611	HKI-0454 Round 2 Line 2
Sample	GG6	ERS20276192	SAMEA115755612	HKI-0454 Round 2 Line 3
Sample	GG7	ERS20276193	SAMEA115755613	HKI-0454 Round 2 Line 4
Sample	GG8	ERS20276194	SAMEA115755614	HKI-0454 Round 2 Line 5
Sample	GG9	ERS20276195	SAMEA115755615	HKI-0454 Round 2 Line 6
Sample	GG10	ERS20276196	SAMEA115755616	HKI-0454 Round 2 Line 7
Sample	GG11	ERS20276197	SAMEA115755617	HKI-0454 Round 2 Line 8
Sample	GG12	ERS20276198	SAMEA115755618	HKI-0454 Round 2 Line 9
Sample	GG13	ERS20276199	SAMEA115755619	HKI-0454 Round 2 Line 10
Sample	GG14	ERS20276200	SAMEA115755620	HKI-0454 Round 1 common ancestor
Sample	R0001	ERS20276201	SAMEA115755621	ATCC62417 strain NH stock
Sample	R0002	ERS20276202	SAMEA115755622	CBS 631.82 strain EH Round 1 common ancestor
Sample	R0003	ERS20276203	SAMEA115755623	CBS 631.82 Round 7 Line 1
Sample	R0004	ERS20276204	SAMEA115755624	CBS 631.82 Round 7 Line 2
Sample	R0005	ERS20276205	SAMEA115755625	CBS 631.82 Round 7 Line 3
Sample	R0006	ERS20276206	SAMEA115755626	CBS 631.82 Round 7 Line 5
Sample	R0007	ERS20276207	SAMEA115755627	CBS 631.82 Round 7 Line 6
Sample	R0008	ERS20276208	SAMEA115755628	CBS 631.82 Round 7 Line 7
Sample	R0009	ERS20276209	SAMEA115755629	CBS 631.82 Round 7 Line 8
Sample	R0010	ERS20276210	SAMEA115755630	CBS 631.82 Round 7 Line 9
Sample	R0011	ERS20276211	SAMEA115755631	CBS 631.82 Round 7 Line 10
Sample	R0012	ERS20276212	SAMEA115755632	CBS 631.82 Round 2 Line 4
Sample	R0013	ERS20276213	SAMEA115755633	CBS 631.82 Round 3 Line 4
Sample	R0014	ERS20276214	SAMEA115755634	CBS 631.82 Round 4 Line 4
Sample	R0015	ERS20276215	SAMEA115755635	CBS 631.82 Round 5 Line 4
Sample	R0016	ERS20276216	SAMEA115755636	CBS 631.82 Round 6 Line 4
Sample	R0017	ERS20276217	SAMEA115755637	CBS 631.82 Round 8 Line 4
Sample	R0018	ERS20276218	SAMEA115755638	CBS 631.82 Round 9 Line 4
Sample	R0019	ERS20276219	SAMEA115755639	CBS 631.82 Round 10 Line 4
Sample	R0020	ERS20276220	SAMEA115755640	CBS 631.82 Round 10 Line 2
Sample	R0021	ERS20276221	SAMEA115755641	CBS 631.82 Round 10 Line 7
Sample	R0022	ERS20276222	SAMEA115755642	CBS 631.82 Round 10 Pooled
Reads	G4_01	ERX12759410	ERR13388646	HKI-0454 starting material «B_{Anc}»
Reads	G4_02	ERX12759419	ERR13388655	HKI-0454 Round 10 Line 2
Reads	G4_03	ERX12759420	ERR13388656	HKI-0454 Round 10 Line 4
Reads	G4_04	ERX12759421	ERR13388657	HKI-0454 Round 10 Line 7
Reads	G4_05	ERX12759422	ERR13388658	HKI-0454 Round 10 Pooled
Reads	G4_06	ERX12759423	ERR13388659	HKI-0454 Round 7 Line 1
Reads	G4_07	ERX12759468	ERR13388704	HKI-0454 Round 7 Line 2
Reads	G4_08	ERX12759469	ERR13388705	HKI-0454 Round 7 Line 3
Reads	G4_09	ERX12759470	ERR13388706	HKI-0454 Round 7 Line 4
Reads	G4_10	ERX12759471	ERR13388707	HKI-0454 Round 7 Line 5
Reads	G4_11	ERX12759472	ERR13388708	HKI-0454 Round 7 Line 6

Reads	G4_12	ERX12759473	ERR13388709	HKI-0454 Round 7 Line 7
Reads	G4_13	ERX12759474	ERR13388710	HKI-0454 Round 7 Line 8
Reads	G4_14	ERX12759475	ERR13388711	HKI-0454 Round 7 Line 9
Reads	G4_15	ERX12759476	ERR13388712	HKI-0454 Round 7 Line 10
Reads	G4_16	ERX12759477	ERR13388713	HKI-0454 Round 3 Line 4
Reads	G4_17	ERX12759478	ERR13388714	HKI-0454 Round 4 Line 4
Reads	G4_18	ERX12759479	ERR13388715	HKI-0454 Round 5 Line 4
Reads	G4_19	ERX12759480	ERR13388716	HKI-0454 Round 6 Line 4
Reads	G4_20	ERX12759481	ERR13388717	HKI-0454 Round 7 Line 4
Reads	G4_21	ERX12759482	ERR13388718	HKI-0454 Round 8 Line 4
Reads	G4_22	ERX12759483	ERR13388719	HKI-0454 Round 9 Line 4
Reads	GG10	ERX12759490	ERR13388726	HKI-0454 Round 2 Line 7
Reads	GG11	ERX12759491	ERR13388727	HKI-0454 Round 2 Line 8
Reads	GG12	ERX12759513	ERR13388749	HKI-0454 Round 2 Line 9
Reads	GG13	ERX12759514	ERR13388750	HKI-0454 Round 2 Line 10
Reads	GG14	ERX12759515	ERR13388751	HKI-0454 Round 1 common ancestor
Reads	GG4	ERX12759484	ERR13388720	HKI-0454 Round 2 Line 1
Reads	GG5	ERX12759485	ERR13388721	HKI-0454 Round 2 Line 2
Reads	GG6	ERX12759486	ERR13388722	HKI-0454 Round 2 Line 3
Reads	GG7	ERX12759487	ERR13388723	HKI-0454 Round 2 Line 4
Reads	GG8	ERX12759488	ERR13388724	HKI-0454 Round 2 Line 5
Reads	GG9	ERX12759489	ERR13388725	HKI-0454 Round 2 Line 6
Reads	R0001	ERX12759526	ERR13388762	ATCC62417 strain NH stock
Reads	R0002	ERX12759531	ERR13388767	CBS 631.82 strain EH Round 1 common ancestor
Reads	R0003	ERX12759557	ERR13388793	CBS 631.82 Round 7 Line 1
Reads	R0004	ERX12759558	ERR13388794	CBS 631.82 Round 7 Line 2
Reads	R0005	ERX12759559	ERR13388795	CBS 631.82 Round 7 Line 3
Reads	R0006	ERX12759560	ERR13388796	CBS 631.82 Round 7 Line 5
Reads	R0007	ERX12759561	ERR13388797	CBS 631.82 Round 7 Line 6
Reads	R0008	ERX12759562	ERR13388798	CBS 631.82 Round 7 Line 7
Reads	R0009	ERX12759563	ERR13388799	CBS 631.82 Round 7 Line 8
Reads	R0010	ERX12759564	ERR13388800	CBS 631.82 Round 7 Line 9
Reads	R0011	ERX12759565	ERR13388801	CBS 631.82 Round 7 Line 10
Reads	R0012	ERX12759567	ERR13388803	CBS 631.82 Round 2 Line 4
Reads	R0013	ERX12759568	ERR13388804	CBS 631.82 Round 3 Line 4
Reads	R0014	ERX12759573	ERR13388809	CBS 631.82 Round 4 Line 4
Reads	R0015	ERX12759574	ERR13388810	CBS 631.82 Round 5 Line 4
Reads	R0016	ERX12759575	ERR13388811	CBS 631.82 Round 6 Line 4
Reads	R0017	ERX12759576	ERR13388812	CBS 631.82 Round 8 Line 4
Reads	R0018	ERX12759577	ERR13388813	CBS 631.82 Round 9 Line 4
Reads	R0019	ERX12759578	ERR13388814	CBS 631.82 Round 10 Line 4
Reads	R0020	ERX12759580	ERR13388816	CBS 631.82 Round 10 Line 2
Reads	R0021	ERX12759581	ERR13388817	CBS 631.82 Round 10 Line 7
Reads	R0022	ERX12759582	ERR13388818	CBS 631.82 Round 10 Pooled
(long)Reads	R0001	ERX12759609	ERR13388845	ATCC62417 strain NH stock
(long)Reads	R0002	ERX12759610	ERR13388846	CBS 631.82 strain EH Round 1 common ancestor
Genome	R0002	ERZ24825341		CBS 631.82 strain EH Round 1 common ancestor
