# Supplementary material for: Inducing novel endosymbioses by implanting bacteria in fungi
Source: Nature. 2024 Oct 2;635(8038):415–22. doi: 10.1038/s41586-024-08010-x (PMC11560845; doi:10.1038/s41586-024-08010-x)
Supplement: Supplementary file 2 — Reporting Summary [file 41586_2024_8010_MOESM2_ESM.pdf]

nature portfolio

Corresponding author(s): Julia A. Vorholt

Last updated by author(s): Aug 8, 2024

Reporting Summary

Nature Portfolio wishes to improve the reproducibility of the work that we publish. This form provides structure for consistency and transparency in reporting. For further information on Nature Portfolio policies, see our Editorial Policies and the Editorial Policy Checklist.

Statistics

For all statistical analyses, confirm that the following items are present in the figure legend, table legend, main text, or Methods section.

n/a	Confirmed	
☐	☒	The exact sample size (*n*) for each experimental group/condition, given as a discrete number and unit of measurement
☐	☒	A statement on whether measurements were taken from distinct samples or whether the same sample was measured repeatedly
☐	☒	The statistical test(s) used AND whether they are one- or two-sided<br>*Only common tests should be described solely by name; describe more complex techniques in the Methods section.*
☒	☐	A description of all covariates tested
☒	☐	A description of any assumptions or corrections, such as tests of normality and adjustment for multiple comparisons
☐	☒	A full description of the statistical parameters including central tendency (e.g. means) or other basic estimates (e.g. regression coefficient) AND variation (e.g. standard deviation) or associated estimates of uncertainty (e.g. confidence intervals)
☐	☒	For null hypothesis testing, the test statistic (e.g. *F*, *t*, *r*) with confidence intervals, effect sizes, degrees of freedom and *P* value noted<br>*Give P values as exact values whenever suitable.*
☒	☐	For Bayesian analysis, information on the choice of priors and Markov chain Monte Carlo settings
☒	☐	For hierarchical and complex designs, identification of the appropriate level for tests and full reporting of outcomes
☒	☐	Estimates of effect sizes (e.g. Cohen's *d*, Pearson's *r*), indicating how they were calculated

Our web collection on statistics for biologists contains articles on many of the points above.

Software and code

Policy information about availability of computer code

Data collection	Data collection as described in the methods section, including VisiView v4.4.0.8
Data analysis	Data analysis as described in the methods section, including FlowJo v10, Flye v2.9.2, BUSCO v5.4.7, BRAKER v3.0.6, BBTools v 38.18, Snippy, bcftools, bcftools-isec, BWA-MEM v0.7, GATK4 v4.2, SnpEff, InterProScan, STRING, Matlab2018a, GraphPad Prism v9.0.0, Fiji 2, iMovie v3.0, GraphPad Prism 9, Adobe Illustrator 2020, BioRender.com and the STRING database: https://string-db.org/. Collected data available under 10.5281/zenodo.12189101, 10.5281/zenodo.12518583, 10.5281/zenodo.13309607, and PRJEB76713 (ENA).

For manuscripts utilizing custom algorithms or software that are central to the research but not yet described in published literature, software must be made available to editors and reviewers. We strongly encourage code deposition in a community repository (e.g. GitHub). See the Nature Portfolio guidelines for submitting code & software for further information.

Data

Policy information about availability of data

All manuscripts must include a data availability statement. This statement should provide the following information, where applicable:

- Accession codes, unique identifiers, or web links for publicly available datasets
- A description of any restrictions on data availability
- For clinical datasets or third party data, please ensure that the statement adheres to our policy

The data availability statement contains the DOIs for the repositories where the data is stored. The repositories will be made public before publication.

Research involving human participants, their data, or biological material

Policy information about studies with human participants or human data. See also policy information about sex, gender (identity/presentation), and sexual orientation and race, ethnicity and racism.

Reporting on sex and gender	N/A
Reporting on race, ethnicity, or other socially relevant groupings	N/A
Population characteristics	N/A
Recruitment	N/A
Ethics oversight	N/A

Note that full information on the approval of the study protocol must also be provided in the manuscript.

Field-specific reporting

Please select the one below that is the best fit for your research. If you are not sure, read the appropriate sections before making your selection.

☒ Life sciences ☐ Behavioural & social sciences ☐ Ecological, evolutionary & environmental sciences

For a reference copy of the document with all sections, see nature.com/documents/nr-reporting-summary-flat.pdf

Life sciences study design

All studies must disclose on these points even when the disclosure is negative.

Sample size	Sample sizes for bacteria injections were kept to n=3 per condition due to clear readout of fungal and bacterial viability and colonization and large effect sizes. For adaptive laboratory evolution, sample size of 10 lines was chosen because multiple lines increase the chance for succesful adaptation and increase the chance for allowing comparisons of affected areas in the genome, even though any single line can yield meaningful results by itself. As adaptive laboratory evolution is labor intensive, a trade-off between maximised results and practicality was made at 10 lines.
Data exclusions	No data was excluded in the analyses that fulfilled the set standards and thresholds. For bacterial injection into the fungus, injections not resulting in a viable fungal germling with visible intracellular bacteria were excluded.
Replication	Successfull bacterial injections were performed in at least triplicates. The adaptive laboratory evolution experiment followed dynamics observed during a pre-trial and was performed in ten lines which showed generally similar trends but also variability in effect sizes. The adaptations by the organisms may not be identically replicated due to the inherent stochasticity in evolutionary experiments.
Randomization	Samples were not randomized as the aim of the study was to describe and characterize effects which could not be foreseen.
Blinding	Investigators were not blinded due to the study following adaptations over time where negative controls are not applicable. Analysis of collected data between samples followed strictly objective criteria.

Reporting for specific materials, systems and methods

We require information from authors about some types of materials, experimental systems and methods used in many studies. Here, indicate whether each material, system or method listed is relevant to your study. If you are not sure if a list item applies to your research, read the appropriate section before selecting a response.

Materials & experimental systems

n/a	Involved in the study	
☒	☐	Antibodies
☒	☐	Eukaryotic cell lines
☒	☐	Palaeontology and archaeology
☒	☐	Animals and other organisms
☒	☐	Clinical data
☒	☐	Dual use research of concern
☒	☐	Plants

Methods

n/a	Involved in the study	
☒	☐	ChIP-seq
☐	☒	Flow cytometry
☒	☐	MRI-based neuroimaging

Plants

Seed stocks: N/A

Novel plant genotypes: N/A

Authentication: N/A

Flow Cytometry

Plots

Confirm that:

☒ The axis labels state the marker and fluorochrome used (e.g. CD4-FITC).

☒ The axis scales are clearly visible. Include numbers along axes only for bottom left plot of group (a 'group' is an analysis of identical markers).

☒ All plots are contour plots with outliers or pseudocolor plots.

☒ A numerical value for number of cells or percentage (with statistics) is provided.

Methodology

Sample preparation: Spores were harvested 6±1 days after injection or plating from spores. Spore solution (8.5% NaCl, 1% Tween20) was added to plates (16 mL for square plates, 12 mL for round plates), and the spores thoroughly detached using a spatula. Remaining mycelium was clumped up and gently pressed with the spatula to release the spore solution from the mycelium. The spore solution was filtered through a 10 µm CellTrics filter (Sysmex, Germany). Spores were washed three times with 1 mL Hepes2 (8000 rcf, 2 min.) and stored at 4 °C ON for FACS.

Instrument: FACSAria Fusion BSL2 Cell sorter (BD, US).

Software: FlowJo v10 (BD, US)

Cell population abundance: During determination of germination rate of positive spores, per sample 5 germlings were checked under the microscope to be populated with GFP-labeled bacteria. If one germling was not positive, another 5 germlings were checked. No sample had to be dismisssed due to more than 2/10 not being colonized by labeled bacteria. The abundance of positive spores varied greatly throughout the experiments, as it was one of the main features studied.

Gating strategy: Single spores were selected using SSC-A/FSC-A and FSC-H/FSC-A gates. Colonization by bacteria was checked using an SSC-A/eGFP-A gate. A gating strategy figure is included in the SI. The whole dataset is available on Zenodo under following link: 10.5281/zenodo.12189101

☒ Tick this box to confirm that a figure exemplifying the gating strategy is provided in the Supplementary Information.
