# Supplementary figures and images for: Inducing novel endosymbioses by implanting bacteria in fungi
Source: Nature. 2024 Oct 2;635(8038):415–22. doi: 10.1038/s41586-024-08010-x (PMC11560845; doi:10.1038/s41586-024-08010-x)

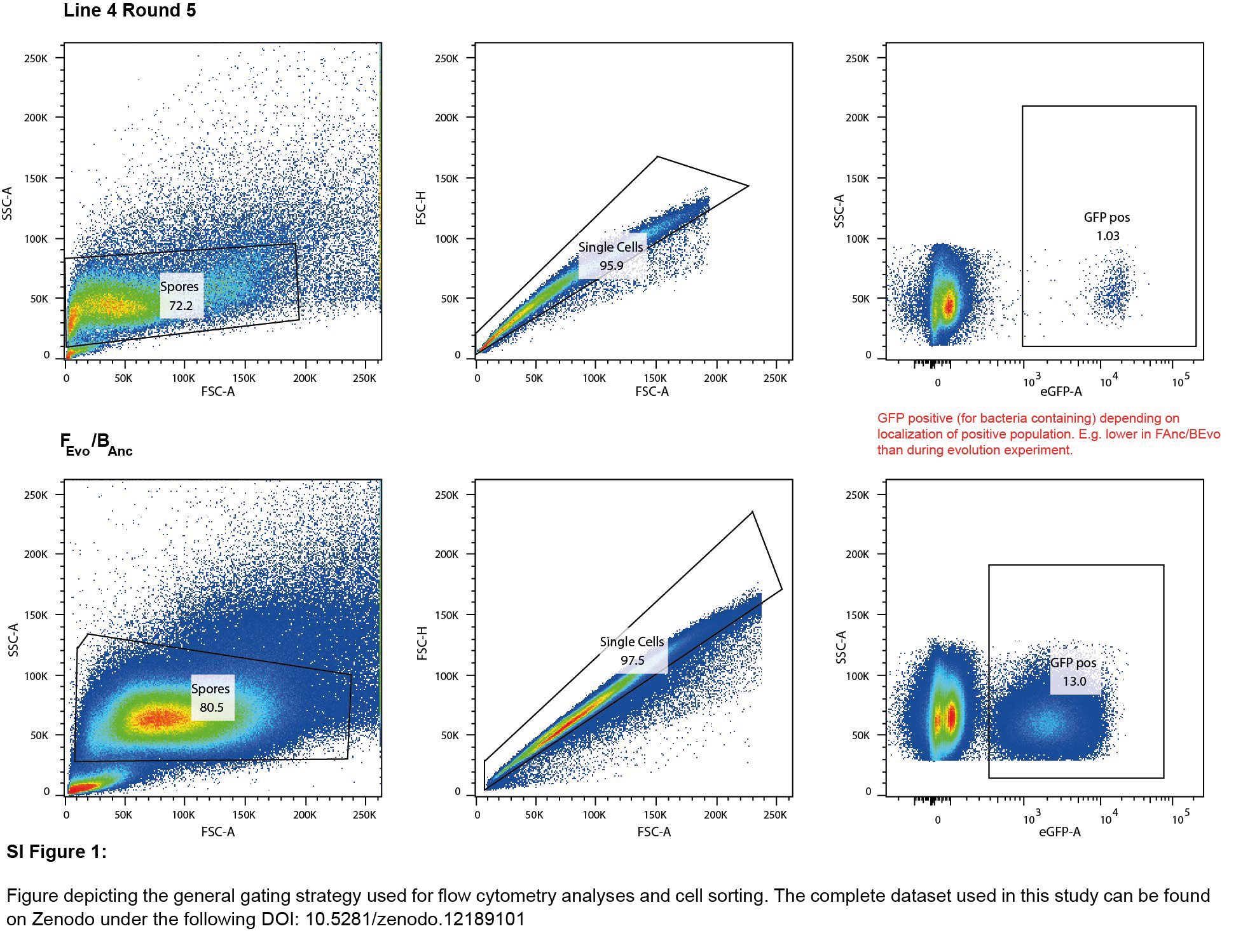

Supplement: Supplementary file 4 — The general gating strategy used for flow cytometry analyses and cell sorting. The complete dataset used in this study is available on Zenodo at https://doi.org/10.5281/zenodo.12189101. [file 41586_2024_8010_MOESM4_ESM.jpg]

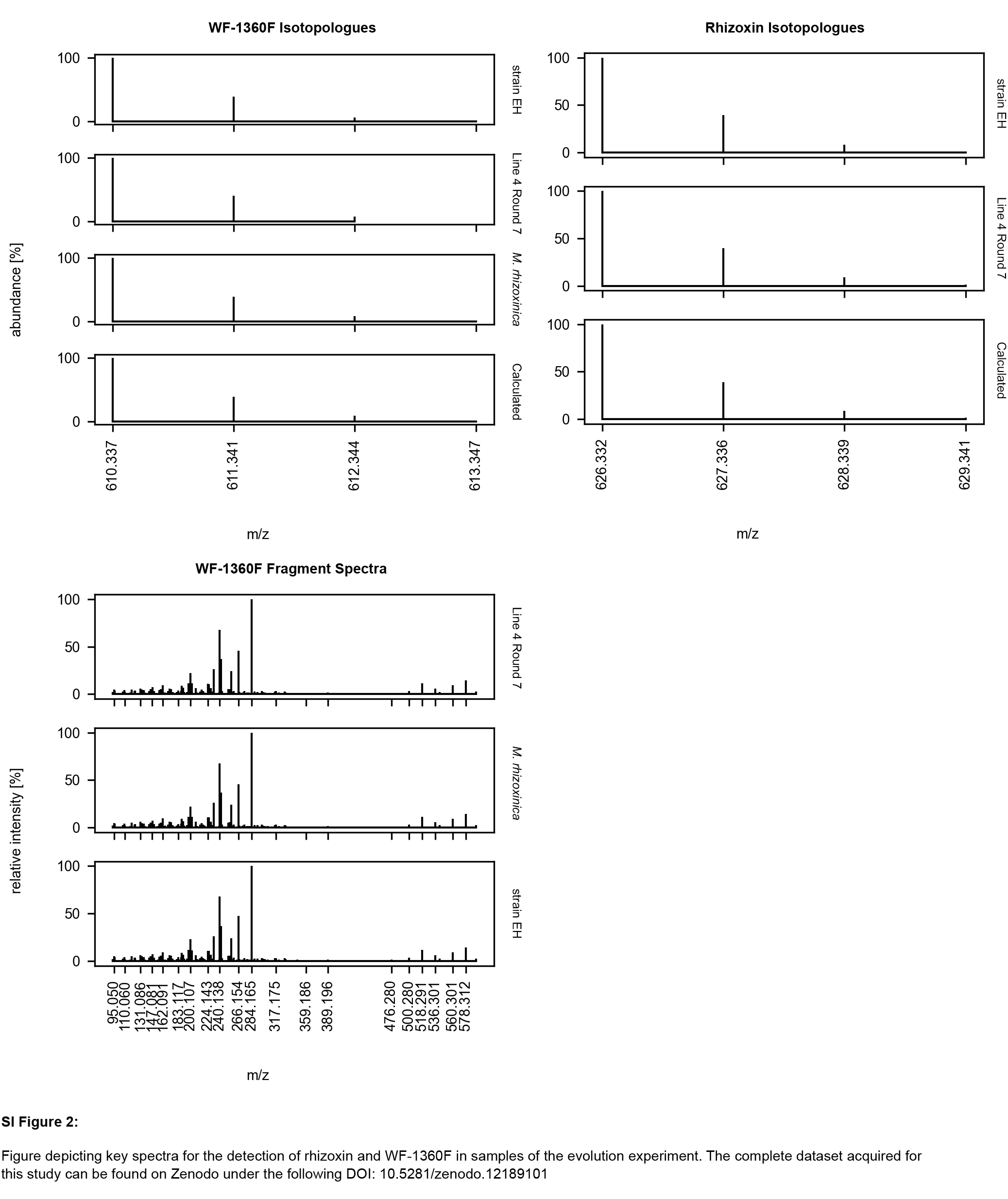

Supplement: Supplementary file 5 — Key spectra for the detection of rhizoxin and WF-1360F in samples of the evolution experiment. The complete dataset acquired for this study is available on Zenodo at https://doi.org/10.5281/zenodo.12189101. [file 41586_2024_8010_MOESM5_ESM.jpg]
